# The Falciform Ligament for Mesenteric and Portal Vein Reconstruction in Local Advanced Pancreatic Tumor: A Surgical Guide and Single-Center Experience

**DOI:** 10.1155/2018/2943879

**Published:** 2018-10-01

**Authors:** T. Malinka, F. Klein, T. Denecke, U. Pelzer, J. Pratschke, M. Bahra

**Affiliations:** ^1^Department of Surgery, Charité Campus Mitte and Charité Campus Virchow Klinikum, Charité-Universitätsmedizin Berlin, Berlin, Germany; ^2^Department of Radiology, Charité Campus Virchow Klinikum, Charité-Universitätsmedizin Berlin, Berlin, Germany; ^3^Department of Hematology/Oncology/Tumor Immunology, Campus Virchow Klinikum, Charité-Universitätsmedizin Berlin, Berlin, Germany

## Abstract

**Background:**

Since local tumor infiltration to the mesenteric-portal axis might represent a challenging assignment for curative intended resectability during pancreatic surgery, appropriate techniques for venous reconstruction are essential. In this study, we acknowledge the falciform ligament as a feasible and convenient substitute for mesenteric and portal vein reconstruction with high reliability and patency for local advanced pancreatic tumor.

**Methods:**

A retrospective single-center analysis. Between June 2017 and January 2018, a total of eleven consecutive patients underwent pancreatic resections with venous reconstruction using falciform ligament. Among them, venous resection was performed in nine cases by wedge and in two cases by full segment. Patency rates and perioperative details were reviewed.

**Results:**

Mean clamping time of the mesenteric-portal blood flow was 34 min, while perioperative mortality rate was 0%. By means of Duplex ultrasonography, nine patients were shown to be patent on the day of discharge, while two cases revealed an entire occlusion of the mesenteric-portal axis. Orthograde flow demonstrated a mean value of 34 cm/s. All patent grafts on discharge revealed persistent patency within various follow-up assessments.

**Conclusion:**

The falciform ligament appears to be a feasible and reliable autologous tissue for venous blood flow reconstruction with high postoperative patency. Especially the possibility of customizing graft dimensions to the individual needs based on local findings allows an optimal size matching of the conduit. The risk of stenosis and/or segmental occlusion may thus be further reduced.

## 1. Introduction

Pancreatic ductal adenocarcinoma is a common malignancy of the gastrointestinal tract and incidences are unfortunately evolving [1]. Despite continuous advancements in interdisciplinary treatment concepts, the majority of patients are still diagnosed at advanced tumor stages and overall long-term prognosis still remains limited [2]. As entire surgical tumor removal remains the best chance for disease-free and long-term survival, recent improvements in surgical expertise have thus been focused on increasing surgical radicality and margin-negative resections rates [3]. While hepatic metastasis, peritoneal carcinomatosis, and/or invasion of major vessels were traditionally considered as parameters for nonresectability, progress in surgical techniques has, however, lead to a debate to redefine modern resection criteria [4, 5]. Nowadays multivisceral resections (MVR) may be considered as feasible and reliable procedures with decreasing complication rates for selected patients [6]. According to this continuous progress, resections of the superior mesenteric vein (SMV), portal vein (PV), or coeliac axis are considered as a safe and reliable technique when performed at high-volume centers [7, 8]. Furthermore, recent studies have shown that survival rates for patients undergoing pancreatectomy with venous reconstruction were comparable to those undergoing pancreatectomy exclusively [9, 10]. However, in case of vascular resection, suitable reconstruction techniques are demanded in order to ensure unimpaired blood flow. When primary direct closure of the venous vessel is not feasible to restore continuity, complex segmental reconstruction, requiring autologous tissue or synthetic materials, is essential [8, 11, 12]. Previously we described bovine pericardium as an innovative and feasible option for venous reconstruction after pancreatic resection [13]. However, autologous materials appear most suitable to maintain venous blood flow continuity and therefore the retrieval of the most appropriate localization for withdrawal is still challenging [14, 15]. The falciform ligament is a broad and thin fold of the peritoneum, attaching the liver to the anterior parietal and diaphragmatic peritoneum, also separating the left and right lobe of the liver. Meaning “sickle-shaped”, from Latin, it is a remnant of the embryonic ventral mesentery with its base being directed downward and backward and its apex upward and backward [16, 17]. Both sides are covered by layers of mesothelial cells and can become canalized in cases of portal hypertension [18, 19]. We herewith report on our initial experience with the falciform ligament as an autologous graft for portal and superior mesenteric vein reconstruction in extended pancreatic resections and provide a surgical guide based on our perioperative outcomes.

## 2. Methods

### 2.1. Patients' Inclusion Criteria and Data Collection

This was a retrospective single-center analysis conducted in a tertiary referral center for pancreatic surgery. Standard preoperative clinical diagnostics included a physical examination and routine laboratory testing. Computed tomography (CT) and/or magnet resonance imaging (MRI) were routinely used as radiological diagnostic tools to analyze tumor diagnosis as well as the stage of the disease. Indications for pancreatic resections were endorsed in an interdisciplinary consensus meeting. All included operations were performed by experienced pancreatic surgeons at the study site and all oncological procedures, as well as venous resections, were performed as open surgical procedures according to international standards [20]. There were no minimal-invasive pancreatectomies within the study group. Accordingly, between June 2017 and January 2018, we identified and reviewed an overall of eleven patients who had undergone en bloc tumor resection with simultaneous venous vascular resection and reconstruction of the venous blood continuity using a falciform ligament graft within our study period. Written informed consent was obtained from all patients.

The following data were collected for each patient: demographics (age, gender); underlying diagnosis; surgical procedure; neoadjuvant chemotherapy; results of the final histopathological examination, including TNM classification; operative details such as operation time, clamping time, and intraoperative transfusion; associated vein resection and reconstruction graft within surgical procedure; details of the perioperative course such as postoperative morbidity in terms of postoperative pancreatic fistula (POPF), postpancreatectomy haemorrhage (PPH), and abdominal collection which were all classified according to International Study Group of Pancreatic Fistula (ISGPF) definitions [21, 22]; length of hospital stay (LOS) was calculated from the day of surgery until the day of discharge; patency of vein and results of orthograde flow measurement during Duplex ultrasonography.

### 2.2. Preconditioning, Technique, and Surgical Guide

When preoperative CT or MRI showed tumor involvement of the celiac axis and/or the common hepatic artery (CHA) but no affiliation to the superior mesenteric artery (SMA) or the gastroduodenal artery (GDA), patients were further evaluated for distal celiacopancreatectomy. If examinations revealed eligible conditions for resection, preoperative embolization of the celiac axis and the CHA was performed. Hereby the arterial blood supply to the liver and stomach was enhanced through collateral pathways from the SMA over the pancreaticoduodenal arcades to the GDA, the proper hepatic artery (PHA), the gastroepiploic artery, and the right gastric artery as previously described [23].

At the beginning of the operation, peritoneal metastases were initially excluded by complete exploration of the abdominal cavity. Access to the omental bursa was established by dissection of the gastrocolic ligament. After retraction of the stomach and inspection of the pancreas, local resectability of the lesion and the extent of the resection were determined based on local findings such as vascular and/or another organ infiltration. In cases of underlying malignant disease, a standard lymphadenectomy was performed. Dissection of the pancreas was done by electrocautery, or in cases of pancreatic left resections with a stapling device.

**Figure 1:**
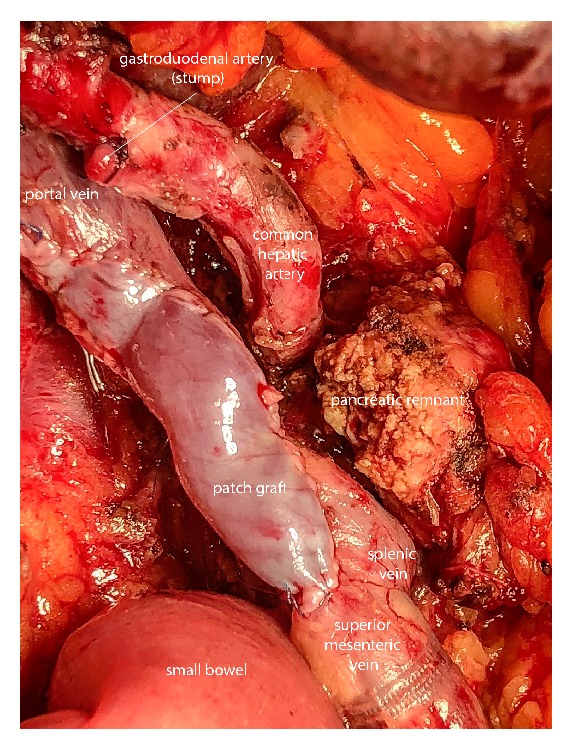
Intraoperative perspective after pylorus-preserving pancreaticoduodenectomy (PPPD) with partial resection of the portal vein and successful reconstruction by patch graft.

**Figure 2:**
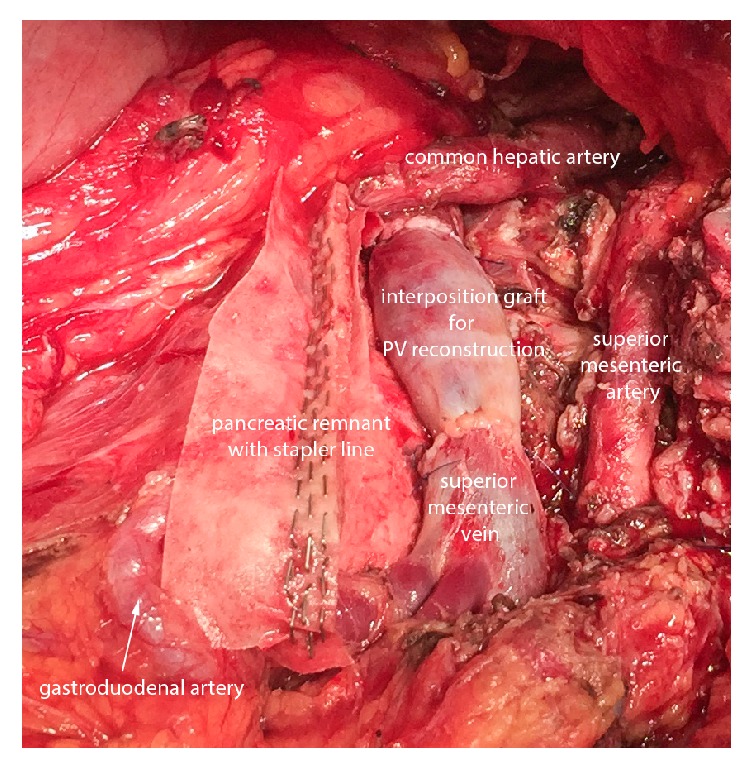
Intraoperative perspective after distal pancreatectomy with en bloc resection of the celiac trunk as well as portal vein segment and successful reconstruction by interposition graft.

In the event of underlying venous infiltration, all veins draining into the impaired segment were clamped and en bloc resection including tumor and vein was conducted. In order to avoid edema in the bowel, cross-clamp time was kept to a minimum. Grafts of the falciform ligament were tailored to the individual needs based on local findings. Therefore, patches from the falciform ligament were retrieved by scissor and kept in saline. In order to shape cylindrical interposition grafts, patches were rolled over a tube and sutured. Preparation of the interposition graft, as well as patch insertion and anastomoses, were all completed by 6.0 Prolene® sutures (Ethicon, Norderstedt, Germany). Cases which required only partial resection of the venous circumference were provided by patch graft (Figure 1), whereas interposition grafts were used to replace segments affected with tumor infiltration of more than half the circumference of the vessel (Figure 2). A growth factor was applied in all anastomosis in order to allow expansion of the suture line as previously described [24]. In case of a pancreatoenteral anastomosis, either a pancreatojejunostomy or a pancreatogastrostomy was performed. Distal closure of the pancreas remnant by stapler was performed using linear stapling devices armed with a 60-mm cartridge (EndoGIA™, Auto- Suture, Covidien) reinforced by a bioabsorbable mesh (SEAMGUARD®, W.L. Gore, Flagstaff, AZ). Every patient received at least one intra-abdominal drain (Degania Silicone Europe GmbH, Regensburg, Germany) to measure postoperative amylase levels and drain output in the postoperative course.

### 2.3. Standard Postoperative Care

**Figure 3:**
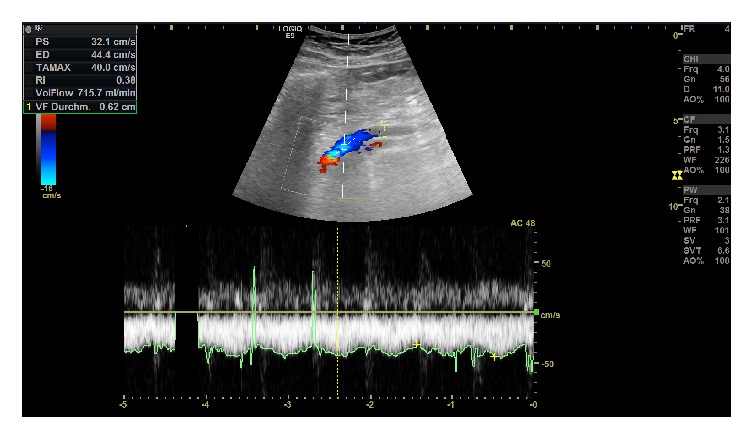
Duplex ultrasonography examination to verify unimpaired portal blood flow in the postoperative course.

Postoperative care was standardized. All patients were monitored for at least one day at a specialized surgical intensive care unit. Anticoagulant therapy after venous reconstruction was performed by continuous administration of unfractionated heparin (UFH) to achieve a PTT of 40-50 seconds for 5 days. Laboratory testing for coagulation variables was performed every 8 hours according to standard ICU care. Afterward, prevention of venous thromboembolism was achieved by subcutaneous application of low molecular weight heparin (LMWH) based on patient's requirement throughout the length of hospital stay. Specific procedure related anticoagulation therapy was discontinued after discharge and only continued if required by additional comorbidities or the underlying diagnose of the individual patient. Portal and superior mesenteric vein blood flow was evaluated by Duplex ultrasonography intraoperative, immediately postoperative as well as on the first and second postoperative day. Final Duplex ultrasonography was performed prior to discharge (Figure 3). If normal hilar portal blood flow along normal liver function was verified, graft patency was assumed sufficient. Contrast-enhanced abdominal computed tomography scanning was not performed regularly but rather on the clinical requirement. Amylase levels were monitored in the serum and in the intraoperatively placed abdominal drains on the second postoperative day. In the absence of signs of a pancreatic fistula, oral food intake was started depending on the clinical presentation and tolerance. The concept of enhanced recovery after surgery (ERAS) has not been applied within the study period. Adjuvant chemotherapy followed depending on the TNM category and clinical situation. All comprehensive procedures were reviewed in an interdisciplinary consensus meeting and patients were seen routinely for follow-up examinations in the outpatient clinic.

## 3. Results

### 3.1. Baseline Characteristics

Between June 2017 and January 2018, a total of eleven consecutive patients underwent pancreatic resections with venous reconstruction by the falciform ligament for extended pancreatic tumor disease at our institution; among them five patients underwent pylorus-preserving pancreaticoduodenectomy (PPPD), two patients received a pylorus preservation total pancreatectomy, two patients received a distal pancreatectomy with splenectomy, and two patients required an Appleby procedure in order to achieve radical tumor removal. There were six males and five females with a mean age of 63 years (43–82) in this group. Three patients received a neoadjuvant chemotherapy, exclusively by FOLFIRINOX, according to an endorsement by an interdisciplinary consensus meeting. Venous resection was performed in nine patients by wedge and in two patients by full segment resection. All venous reconstructions were performed utilizing portions of the falciform ligament. Histological examination showed ductal adenocarcinomas in ten patients, while one patient suffered from an invasive intraductal papillary mucinous carcinoma (IPMC) of the pancreas. In four cases complete resection of tumor tissue was achieved, while in seven cases residual tumor was present microscopically at retroperitoneal resection margins (Table 1).

### 3.2. Perioperative and Postoperative Data

The mean duration of the surgical procedure was 361 min, while mean clamping time of the mesenteric-portal blood flow was 34 min. In seven patients the portal vein was solely infiltrated by tumor, whereas in four cases tumor involvement of the confluence was determined. Nine cases were provided by patch graft, while two patients required an interposition graft due to a segmental tumor infiltration of more than half the circumference of the underlying venous vessel. In six patients a transfusion of red blood cells (RBC) and fresh frozen plasma (FFP) was necessary during the intraoperative course. Mean ICU length of stay and overall hospital stay was 4 (1-12) and 23 (12-59) days, respectively. The perioperative mortality rate was 0%. Two patients developed POPF grade B, while two patients sustained post pancreatectomy haemorrhage. In one case, a bleeding from the anastomosis of the interposition graft occurred and surgical revision was required. The other case demonstrated a bleeding from the gastroduodenal artery, although no pancreatic fistula was observed. Coiling of the stump of the GDA stopped PPH and no surgical intervention was necessary. Both cases of POPF grade B were managed by percutaneous drainage and fistula resolved in the course without further intervention. In one patient, a CT scan revealed an abdominal collection, which was approached by CT guided percutaneous drainage. By means of Duplex ultrasonography, nine patients were shown to be patent on the day of discharge, while two cases revealed an entire occlusion of the mesenteric-portal axis. Both patients presented ascites in the postoperative course and required diuretics. Reopening by interventional approach was unsuccessfully attempted in one patient. Both patients received anticoagulant therapy and could be discharged uneventfully. Orthograde flow demonstrated a mean value of 34 cm/s for patent grafts. All patent grafts on discharge revealed persistent patency within various assessments in our oncological outpatient clinic or direct communication with the general practitioner (Table 2). After discharge, eight patients were included in the study protocol of the Hyperthermia European Adjuvant Trial (HEAT; ClinicalTrials.gov Identifier: NCT01077427) for further treatment, based on the recommendations of the interdisciplinary consensus meeting. Two patients sustained FOLFIRINOX for their adjuvant treatment, while to one patient gemcitabine/nab-paclitaxel was administered (Table 2).

## 4. Discussion

Clinical outcome after pancreatic surgery has improved considerably over the last decades with a consistent reduction of postoperative morbidity and mortality [25]. While previous studies indicated no significant differences in terms of postoperative morbidity and long-term survival after pancreatectomy with mesenteric-portal vein resection, recent studies demonstrated beneficial outcomes and increased resectability rates for patients with underlying pancreatic malignancies [5, 9, 26]. Since local tumor infiltration to the venous blood flow might represent a challenging assignment for curative intended resectability, appropriate techniques for venous reconstruction are essential [27, 28]. In this study, we acknowledge the falciform ligament as a feasible and convenient substitute for mesenteric and portal vein reconstruction with high patency and reliability in extended pancreatic tumor resections.

After venous resection has been performed, rearrangement of the mesenteric-portal axis is essential to ensure unimpaired blood flow continuity. Even though the primary direct closure of the venous vessel is frequently feasible, pancreatic surgeons partially encounter complex situations requiring additional tissue for reconstruction [14, 29, 30]. Various autologous grafts have been described in the literature, including the great saphenous vein, internal jugular vein, femoral vein, left renal vein, or gonadal veins [11, 12, 31–33]. Harvesting of these grafts may, however, be accompanied by relevant morbidity due to the impermanent into another anatomic region [12, 34, 35]. Postoperative edema, increased operative time, increased risk of bleeding or size mismatches resulting in deficient portal inflow with an increased risk of turbulence that might promote clotting of the graft are frequently reported [8]. In addition, synthetic grafts composed of polytetrafluoroethylene (PTFE) or polyethylene terephthalate (Dacron) contain a seriously substandard risk of thrombosis and infection and an elongated anticoagulation therapy postoperative is demanded [36]. Consequently, also synthetic grafts are not commonly recommended as grafts for mesenteric and portal vein reconstruction and therefore the most suitable tissue still remains to be identified [28, 37].

Previously we described bovine pericardium as a safe and feasible material for mesenteric and portal vein reconstruction during pancreatic surgery and indicated good patency and easy handling [13]. Although the introduced procedure is still frequently implemented in our tertiary referral center for pancreatic surgery, we targeted an even more autologous and cost-effective solution.

The falciform ligament, a remnant of the embryonic ventral mesentery, is a broad and thin fold of the peritoneum that is covered with epithelial layers on both sides featuring several advantages. Within our observation period, we discovered good graft patency, which is comparable with results of other studies using different autologous tissue or interposition grafts compounded by PTFE [32, 37]. However, retrieval, as well as implementation, is much easier and dimensions can be customized to the individual needs based on intraoperative findings. Furthermore, due to its autologous nature, the risk of infection and the necessity for long-term anticoagulation therapy postoperative is limited. In addition, cost-effective availability results in verifiable benefits in favor of the autologous falciform ligament. Surprisingly, our results are in contrast to a comparable study also targeting the falciform ligament as a graft for portal and mesenteric vein reconstruction. Zhiying et al. reported good graft patency until two weeks postoperatively, while an increased occlusion rate up two months postoperatively was observed [38]. Interpretations of differences to our findings are very difficult to distinguish and due to the small number of cases within both series, further studies need to trace long-term patency and overall outcome. Nevertheless, information regarding the chosen suture material, as well as placement of a growth factor, has not been depicted within their manuscript and could therefore possibly be accountable for the documented stenosis and occlusion in the follow-up. Although there is a significant heterogeneity regarding anticoagulation after venous reconstruction with pancreatic resection and currently no agreed approach available [36], continuous administration of unfractionated heparin (UFH) to achieve a PTT of 40-50 seconds for 5 days was conducted in our series by contrast. However, a significant impact on occlusion rates in the course remains debatable.

The most frequent complication in our series was the occurrence of a pancreatic fistula, which can be seen as being related to the pancreatic resection itself. However, two patients were consequently affected by PPH demanding further intervention. In one case bleeding occurred from the stump of the gastroduodenal artery, which was controlled by an angiographic procedure, while the other patient was impaired from a direct bleeding from the venous interposition graft due to an occult arrosion of the suture material. According to other series, PPH occurred frequently and appears to be independent of the chosen tissue for venous reconstruction [39].

Microscopic resection margin involvement (R1 resection) was present in 7 patients, of which all involved the retroperitoneal margins. However, superior mesenteric-portal vein resections including tangential resection with a patch or segmental resection with interposition graft revealed margin-free resections at this point in all eleven cases. Accordingly, the falciform ligament appears to be a safe and feasible substitute to achieve venous R0 resections when the tumor cannot be separated from the venous axis.

The present study is, of course, limited by common biases that are mainly due to the retrospective character of this analysis and the heterogonous time specification concerning long-term longevity. Therefore, further studies with increased numbers of cases need to determine observations.

In conclusion, pancreatectomy with resection of the mesenteric-portal axis due to tumor infiltration provides the chance for complete tumor resection. In our series, the falciform ligament appears to be a feasible and reliable autologous tissue for venous blood flow reconstruction with high postoperative patency and low risk of infection. Due to the possibility of customizing graft dimensions to the individual needs based on local findings, optimal size matching of the conduit is feasible and thus the formation of stenosis and segmental occlusion may be less likely. Although rates of patency in our patients are promising, conclusions on longevity still need to be evaluated in further enlarged observations.

## Figures and Tables

**Table 1:** Patient demographics, surgical procedure, and histopathologic results.

**Patient**	**Age/Sex**	**Diagnosis**	**Surgical procedure**	**Neoadjuvant chemotherapy**	**Pathology (TNM)**	**Category**
A	49/M	Ductal adenocarcinoma pancreatic body	Distal pancreatectomy, splenectomy, celiac trunk resection, portal vein wedge resection	No	pT2 pN1 (2/24) L1 V1 Pn1 G3	R1

B	66/M	Ductal adenocarcinoma pancreatic head	PPPD, portal vein wedge resection	No	pT2 pN1 (3/21) L1 V0 Pn1 G2	R1

C	82/M	IPMC	PPPD, portal vein wedge resection	No	pT1c pN0 (0/28) L0 V0 Pn0 G2	R0

D	63/M	Ductal adenocarcinoma pancreatic body	Distal pancreatectomy, splenectomy, celiac trunk resection, portal vein wedge resection	No	pT2 pN0 (0/11) L0 V1 Pn1 G2	R1

E	58/F	Ductal adenocarcinoma pancreatic tail	Distal pancreatectomy, splenectomy, Portal vein segment resection	Yes FOLFIRINOX (6#)	ypT2 ypN0 (0/11) L0 V1 Pn1 G2	R1

F	65/F	Ductal adenocarcinoma pancreatic body	Distal pancreatectomy, splenectomy, Portal vein wedge resection	No	pT2 pN2 (5/48) L0 V1 Pn1 G3	R1

G	70/M	Ductal adenocarcinoma pancreatic head	Pylorus preservation pancreatectomy, portal vein wedge resection	Yes FOLFIRINOX (10#)	ypT2 ypN2 (4/24) L0 V0 Pn0 G2	R0

H	63/F	Ductal adenocarcinoma pancreatic head	Pylorus preservation pancreatectomy, portal vein wedge resection	Yes FOLFIRINOX (6#)	ypT3 ypN1 (1/17) L0 V0 Pn1 G2	R1

I	72/F	Ductal adenocarcinoma pancreatic head	PPPD, portal vein wedge resection	No	pT3 pN1(1/12) L0 V1 Pn1 G2	R1

J	43/F	Ductal adenocarcinoma pancreatic head	PPPD, portal vein segment resection	No	pT2 pN0 (0/14) L0 V0 Pn1 G2	R0

K	63/M	Ductal adenocarcinoma pancreatic head	PPPD, portal vein wedge resection	No	pT3 N0 (0/28) R0 L0 V0 Pn1 G3	R0

**Table 2:** Perioperative and postoperative data after pancreatectomy with en bloc venous resection and reconstruction by falciform ligament.

**Patient**	**Operative time (min)**	**Involved vein**	**Clamping (min)**	**Type of graft**	**RBC/FFP**	**Hospital/ ICU length of stay (day)**	**Perioperative complications**	**Patency ** **of Vein prior to discharge**	**Orthograde flow**	**Adjuvant chemotherapy**	**Diabetes mellitus**	**Outcome Patency**
A	300	PV	35	Patch	3/6	37/5	Pancreatic fistula (Bassi B)	Occlusion	n.a.	HEAT	No	Occlusion

B	332	PV	30	Patch	0/0	10/2	None	Patent	Vmax = 27 cm/s	Gemcitabine/nab-paclitaxel	No	Patent (POD 147)

C	410	PV	25	Patch	2/4	20/9	PPH	Patent	Vmax = 32 cm/s	HEAT	Insulin-dependent	Patent (POD 118)

D	368	PV	30	Patch	4/8	28/2	Abdominal collection	Patent	Vmax = 28 cm/s	HEAT	No	Patent (POD 64)

E	336	PV	45	Interposition	0/0	12/1	None	Patent	Vmax = 21 cm/s	HEAT	No	Patent (POD 57)

F	314	Confluens	35	Patch	2/4	59/7	Pancreatic fistula (Bassi B)	Patent	Vmax = 57 cm/s	HEAT	No	Patent (POD 74)

G	368	Confluens	30	Patch	6/12	14/2	None	Patent	Vmax = 35 cm/s	FOLFIRINOX	Insulin-dependent	Patent (POD 88)

H	485	Confluens	30	Patch	4/8	15/2	None	Patent	Vmax = 44 cm/s	FOLFIRINOX	Insulin-dependent	Patent (POD 39)

I	348	PV	35	Patch	0/0	19/2	SSI	Patent	Vmax = 28 cm/s	HEAT	No	Patent (POD 30)

J	371	PV	50	Interposition	0/0	29/12	PPH	Occlusion	n.a.	HEAT	No	Occlusion

K	348	Confluens	30	Patch	0/0	18/2	None	Patent	Vmax = 32 cm/s	HEAT	No	Patent (POD 18)

## Data Availability

The datasets generated and analyzed during the current study are available from the corresponding author on reasonable request.
